# The HIV-1 Rev response element (RRE) adopts alternative conformations that promote different rates of virus replication

**DOI:** 10.1093/nar/gkv313

**Published:** 2015-04-08

**Authors:** Chringma Sherpa, Jason W. Rausch, Stuart F.J. Le Grice, Marie-Louise Hammarskjold, David Rekosh

**Affiliations:** 1 Myles H. Thaler Center for AIDS and Human Retrovirus Research, Department of Microbiology, Immunology, and Cancer Biology, University of Virginia, Charlottesville, VA 22908, USA; 2 Center for Cancer Research, National Cancer Institute, NIH, Frederick, MD 21702, USA

## Abstract

The HIV Rev protein forms a complex with a 351 nucleotide sequence present in unspliced and incompletely spliced human immunodeficiency virus (HIV) mRNAs, the Rev response element (RRE), to recruit the cellular nuclear export receptor Crm1 and Ran-GTP. This complex facilitates nucleo-cytoplasmic export of these mRNAs. The precise secondary structure of the HIV-1 RRE has been controversial, since studies have reported alternative structures comprising either four or five stem-loops. The published structures differ only in regions that lie outside of the primary Rev binding site. Using in-gel SHAPE, we have now determined that the wt NL4-3 RRE exists as a mixture of both structures. To assess functional differences between these RRE ‘conformers’, we created conformationally locked mutants by site-directed mutagenesis. Using subgenomic reporters, as well as HIV replication assays, we demonstrate that the five stem-loop form of the RRE promotes greater functional Rev/RRE activity compared to the four stem-loop counterpart.

## SIGNIFICANCE

The Rev–RRE axis of human immunodeficiency virus (HIV) gene regulation is a unique post-transcriptional system that is essential for HIV RNA trafficking and viral replication. The initial step in this pathway is the binding of Rev to an RNA element (RRE) found the HIV genomic RNA and some HIV mRNAs. Our results demonstrate that the RRE can modulate HIV replication kinetics by adopting different conformations that alter the nature of the complex formed and the rate by which the virus replicates. This previously undescribed mechanism could regulate HIV replication in response to various immunological or cellular cues.

## INTRODUCTION

In an infected cell, integrated HIV proviral DNA is transcribed to produce a primary transcript that either remains unspliced or is alternatively spliced into multiple mRNA species that retain or lack introns (1,2). These mRNAs are all exported to the cytoplasm, where they are packaged as the viral genome or translated into viral proteins, despite the fact that eukaryotic cells have several ‘checkpoints’ that would normally restrict export and expression of the mRNAs that retain introns (3–5). This is due to the fact that HIV and other retroviruses have evolved specific mechanisms to overcome these restrictions.

Most complex retroviruses, including HIV (6–8), HTLV (9,10), MMTV (11), EIAV(12,13) and Jaagsiekte Sheep virus (14,15), encode a regulatory protein that interacts with a *cis*-acting element in the viral transcripts to mediate nuclear export and translation of intron-retaining mRNA through the Crm1 pathway. There is little sequence similarity in either the regulatory proteins or the RNA elements across these viruses. In contrast, the simpler retroviruses, such as Avian Leukosis Virus (16), Moloney Leukemia Virus (17,18) and Mason-Pfizer Monkey Virus (19), utilize *cis*-acting elements that interact directly with host cell factors to mediate the export and subsequent translation of intron-retaining mRNAs via the Nxf1 export pathway. Foamy viruses are unique in that they use a cellular RNA-binding protein, HuR, instead of a viral regulatory protein to facilitate nuclear export of the unspliced viral transcripts through the Crm1 pathway (20).

HIV uses the viral Rev protein and its RNA binding partner, the Rev response element (RRE), to overcome the cellular mRNA intron surveillance mechanisms (5,21). The RRE is a *cis*-acting RNA element present in all intron-retaining viral mRNAs. Produced from a fully spliced mRNA, Rev shuttles back into the nucleus and binds specifically to its primary binding site in the RRE (22–26). Additional Rev molecules are recruited by cooperative binding, in a process that involves protein–protein as well as protein–RNA interactions (23,27–35). Rev binding and subsequent oligomerization are essential for Rev/RRE function (29,31,36,37). While the former allows Rev to make specific contacts with the RRE (38), oligomerization increases its affinity for the RRE ∼500-fold (33). Assembly studies, performed under ensemble-average conditions, such as filter binding, gel mobility shift, chemical footprinting, mass spectrometry, fluorescence resonance energy transfer and surface plasma resonance (27–29,39–42), have indicated that Rev binds incrementally to the RRE. A recent single molecule Rev/RRE binding study, using total internal reflection fluorescence microscopy, also confirmed that Rev binds to the RRE in monomeric increments (43). The Rev/RRE complex is then recognized by Crm1 and Ran-GTP, forming an export competent ribonucleoprotein (RNP) complex (44,45).

Despite the wealth of knowledge about Rev/RRE function, the precise secondary structure of the RRE has remained controversial. The RRE is a 351 nt, highly structured RNA region consisting of multiple stem-loops and bulges (30,46). Computational modeling and chemical and enzymatic probing methods, alone or in combination, have been used to investigate the secondary structure of the RRE *in vitro*. Many of these studies (46–48) reported that the RRE forms a five stem-loop (SL) structure, where the 5′ end and the 3′ end of RRE base-pair to form the central base of the RRE—named SL I/I’. Often SL I/I’ opens into a central loop, that branches into four additional SL (SL II, III, IV and V) but a recent study reported a 5 SL structure in which part of the central loop is base paired (49). In contrast, other studies (30,39,42) have suggested that the RRE forms an alternative 4 SL structure that differs structurally in the rearrangement of SL III and IV. In this alternative structure, SL III and IV of the five SL RRE combine to form a single SL III/IV off of the central loop.

The primary Rev binding site on the RRE has been mapped to stem IIB (27,47,50) and stem I/I’ has been shown to provide a secondary site (30,33). However, although there is some evidence that SL III, IV and V contribute to Rev/RRE function (39,51,52), their precise roles are poorly understood and the literature contains conflicting reports. For example, Olsen *et al*. (37) showed that deleting SL III significantly reduced the affinity of Rev for the RRE as measured by a nuclease protection gel retardation assay. In addition, a Rev responsive chloramphenicol-acetyl transferase reporter assay with this mutant showed that the deletion reduced Rev/RRE function 5-fold. In contrast, others (48,53) showed that individual deletion of each SL minimally affected Rev/RRE function in reporter assays but simultaneous deletion of both SL IV and SL V resulted in a 3-fold decrease in function (53). However, in these assays, Rev was significantly overexpressed compared to the levels seen in a viral infection, which could have masked the potential importance of SL III–V. In other studies, mutations predicted to disrupt SL III, IV and V displayed significantly lower Rev/RRE activity (48,51) and oligonucleotides complementary to the apical loop of SL V blocked Rev–RRE function to the same level as those directed to the primary Rev binding site in SL II (51). Interestingly, these oligonucleotides did not block Rev binding to the RRE *in vitro*, but were capable of completely disrupting preformed Rev/RRE complexes. There is also recent work by Bai *et al*. that suggests that the SL I/I’ of the RRE makes long-range tertiary contacts with the certain regions of the central loop, SL III, IV and V forming a compact structure that provides additional Rev binding sites needed to form the final Rev/RRE complex (49).

Previous work from our own groups has shown that single nucleotide changes in RRE SL III–V can overcome resistance to the transdominant negative *rev* protein, Rev M10 (39). Although the mechanism of resistance mediated by these single nucleotide changes remains unknown, we have shown that they induce a rearrangement of SL III, IV and V into an alternative stable structure that closely resembles the 5 SL variant (39). This led us to hypothesize that the 5 SL RRE may convey a selective replication advantage over its 4 SL counterpart. The present study describes experiments that directly test this hypothesis.

## MATERIALS AND METHODS

See Supplemental Data for more detailed information.

### RNA synthesis

RRE RNAs, 232 nucleotides in length, with a 3′ structure cassette were *in vitro* transcribed using the MegaShortScript kit (Ambion). RNAs were gel purified (5% polyacrylamide, 7M urea), eluted, precipitated and stored at −20°C in TE buffer [10 mM Tris (pH 7.6), 0.1 mM EDTA].

### RNA folding

RNAs (∼20 picomoles for gel migration and conventional SHAPE and 75 picomoles for in-gel SHAPE experiments) resuspended in 10 mM Tris (pH 8.0), 100 mM KCl, 0.1 mM EDTA were heated at 85°C for 2 min and slowly cooled to 25°C. The RNAs were incubated in the refolding buffer [70 mM Tris (pH 8.0), 180 mM KCl, 0.3 mM EDTA, 5 mM MgCl_2_,] and, for in-gel SHAPE only, with an additional 5% glycerol, at 37°C for 30 min.

### In-gel SHAPE experiments

Folded RNAs were fractionated on a native 8% polyacrylamide gel containing 5 mM MgCl_2_ at constant 200 V for 22 h at 4°C. For in-gel SHAPE, RRE comformers were separately excised and incubated with 1X TBE buffer containing 10% DMSO and 10 mM NMIA at 37°C for 50 min. Gel-slices were washed with 1X TBE, crushed and electroeluted. In conventional SHAPE, folded RNAs were directly treated with 3 mM NMIA in DMSO. The modified RNAs from these procedures were precipitated, resuspended in water and reverse transcribed. The resulting cDNA fragments were resolved by capillary electrophoresis and subsequent data analysis and representation were performed as previously described (54).

### Rev–RRE gel shift assay

Internally ^32^P-labeled RNAs were prepared by *in-vitro* transcription, DNase I treated, gel (4% polyacrylamide, 5M Urea) purified and eluted overnight in TE buffer. The RNAs were phenol-chloroform purified, ethanol precipitated and resuspended in water. They were then folded by incubation in 50 mM NaCl, 10 mM HEPES/KOH, (pH 7.6), and 2 mM MgCl_2_ at 85°C for 3 min and then at room temperature for 15 min. Approximately 1 nM of the folded RNAs were incubated with serially diluted Rev in the presence of Rev binding buffer [10 mM Hepes/KOH(pH7.8), 20 mM KCL, 2 mM MgCl_2_, 0.5 mM EDTA, 1 mMDTT, 10% glycerol, 5 μg/ml yeast tRNA and 20U RNase inhibitor] in a volume of 10.6 μl on ice for at least 10 min. Rev/RRE complexes were resolved on 4% non-denaturing polyacrylamide gel run at 20mA for 4 h at 4°C. Gels were dried, then visualized and quantified using Molecular Dynamics Phosphorimager and ImageQuant software.

### Rev-dose response assay

This assay was performed as described previously (55) by co-transfecting 293T cells with 2 μg of a GagPol-RRE (351 nt RRE) construct and 0–16 ng Rev expression plasmid. The Rev/RRE activity was measured by quantitating the p24 secreted into the cell medium.

### Hygromycin resistance assay

Transducing viral stocks were produced by co-transfecting 293T cells with 20 μg pTR167Nef- w/ RRE (351 nt RRE), 15 μg pCMVΔR9 and 5 μg pCMVVSV-G using the calcium phosphate method. Serial dilutions of viral stocks were used to infect Hela cells using DEAE dextran. Starting 2 days post transfection, cells were maintained in hygromycin medium (200 μg/ml) until day 14. Hygromycin-resistant colonies were then crystal violet stained and counted.

### Spreading infections

Transfection viral stocks were prepared by transfecting 293T cells with 5 μg of the proviral DNA containing a 351-nt RRE in the Nef position (see Supplemental Information for details of these constructs). For the first infection, SupT1 cells were infected with the p24 equivalent of transfection viral stocks. Every 3–4 days, two-thirds of the culture was replaced with fresh medium and p24 levels in the replaced medium were measured to generate the growth curve. For the second infection, the TCID50 of the infection supernatant from the peak replication day of the first infection was determined as described by Abraha *et al*. (56). Such SupT1 passaged virus at a multiplicity of infection (MOI) of 0.000005 was used to infect fresh SupT1 cells, using DEAE-dextran, and a growth curve was generated as described above.

### Growth competition assay

This assay was mostly performed as previously described (56). SupT1 cells were infected with a 0.000005 MOI of each of the two competing viruses. The viral stocks used were the infection supernatants from the peak replication day of the spreading infection described above. Ten days post infection, viral DNA was isolated and the amount of each proviral DNA in each culture was determined by a heteroduplex tracking assay (56).

## RESULTS

### Evidence for two secondary structures of the wt NL4-3 RRE

**Figure 1.**
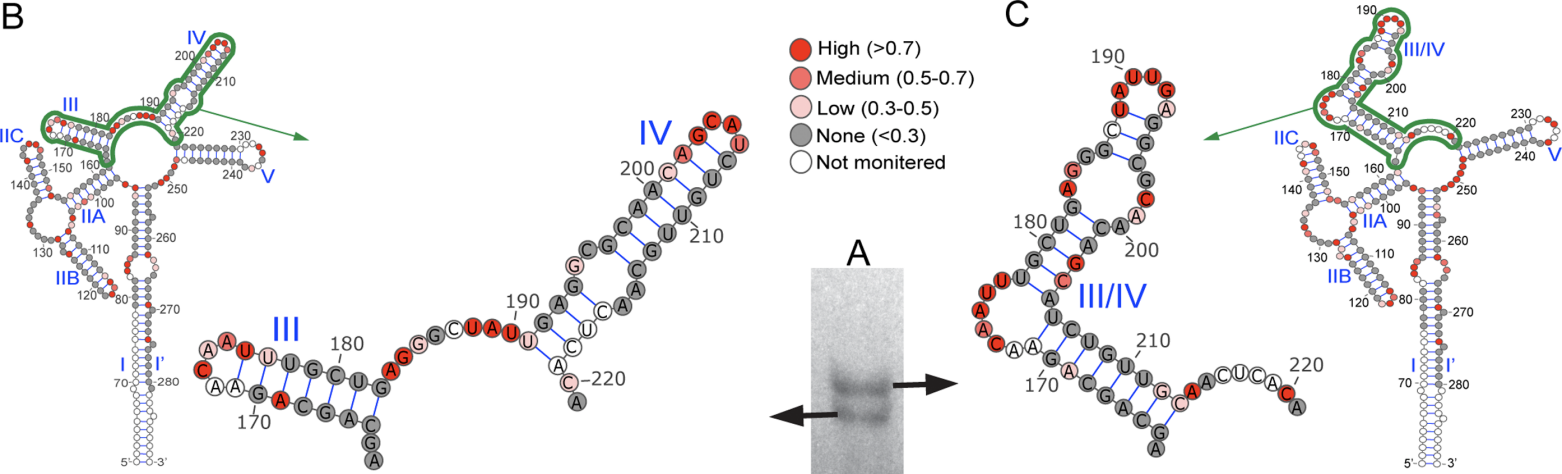
(**A**) Native polyacrylamide gel in which RRE conformers are visualized by UV shadowing. Structures of the SL III and IV regions of the RRE. 5 SL (**B**) and 4 SL (**C**) NL4-3 RRE variants as determined by in-gel SHAPE. Reactivity values of the various residues are color coded according to the key.

The RRE sequence (‘short RRE’ Supplementary Figure S1) used in this*in vitro* study comprises pNL4-3 nucleotides 7760–7992 (GenBank accession no. AF324493). This RRE is functional, although it lacks the lower part of SL I present in the 351 nt RRE structure and it is the form commonly used in gel mobility shift and other studies. It comprises nucleotides 60–292 in the RRE sequence of Charpentier *et al*. (42). To re-examine its secondary structure, the RRE, appended with a short primer binding cassette, was synthesized *in vitro*, refolded and analyzed by non-denaturing polyacrylamide electrophoresis. Figure 1A shows that, under prolonged electrophoresis, the RRE migrated as two bands. To examine the structure of each RNA, the two bands were excised individually and subjected to in-gel SHAPE (57) using NMIA as the acylating reagent. Reactivity values of 17 nt at the 5′ end and 11 nt at the 3′ end of the RRE were unquantifiable due to the loss of signal resolution. Of the remaining RRE nucleotides, reactivity values were obtained for > 92%. Almost all unscored nucleotides (216–219, 171/172, 129–131,143/144, 231/232) were consistently unquantifiable, possibly reflecting structure-induced pausing of reverse transcriptase at these bases. Figure 1B and C show the most energetically favorable structures assumed by RRE nucleotides 163–221 in the two excised RNAs as predicted by RNAStructure (v5.3) using the respective sets of SHAPE reactivity constraints. Reactivity values for this subset of RRE nucleotides are indicated as color-coded circles in these panels, while complete structures/values for each RRE are provided in Supplementary Figure S2. These results indicate that the faster and slower migrating RNAs adopted a 5 SL and 4 SL structure, respectively. We therefore concluded from this analysis that the NL4-3 RRE can exist as an approximately equimolar mixture of the two structures.

### Constructing RRE mutants with altered secondary structures

We next attempted to create mutations that stabilized the 4 SL and 5 SL RRE variants (Supplementary Figures S3–S7). Mutant A was designed to disrupt base pairing at the base of the combined SL III/IV in the 4 SL structure while maintaining base pairing in SL IV in the 5 SL structure, thus favoring the 5 SL configuration. Conversely, Mutant B was expected to disrupt base pairing in both SL III and SL IV of the 5 SL structure while keeping the combined SL III/IV intact, thereby favoring the 4 SL configuration. Mutant C created aberrant base pairing within the combined SL III/IV that could allow it to form a severely altered SL III/IV, but maintain a 4 SL configuration. Mutants D and E were expected to disrupt SL III/IV in the 4 SL structure and SL IV in the 5 SL structure and thus were unlikely to assume either configuration.

**Figure 2.**
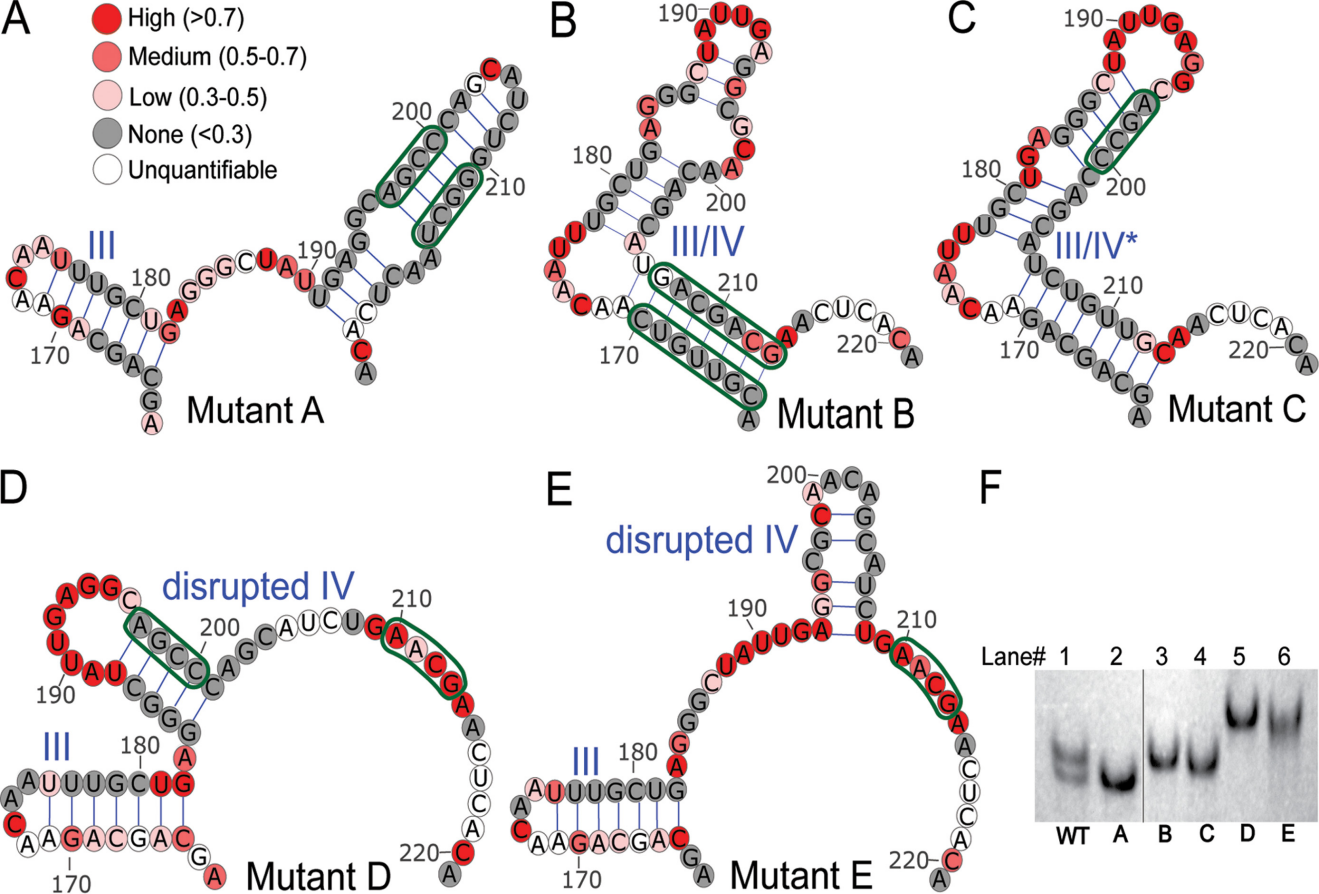
Structures of SL III and IV regions of RRE mutants as determined by SHAPE. Panels **A–E** show the various mutant structures. The structures of the other regions of the RRE were identical to the wt and are shown in Supplementary Figures S2–S7. (**F**) Migration of wt and mutant RREs over an 8% native polyacrylamide gel visualized by UV shadowing. Samples were analyzed on the same gel. The vertical line between lanes 2 and 3 indicates that a lane not relevant to this work has been deleted.

The secondary structures of mutant RREs were next examined to verify our predictions. Mutant and wt RRE RNAs appended with the same 3′ tag were prepared by *in vitro* transcription, refolded and fractionated by non-denaturing polyacrylamide gel electrophoresis. Figure 2F shows that each RRE mutant migrated as a homogeneous species. Importantly, Mutant A RRE migrated to a position coincident with the 5 SL wt RRE, whereas Mutant B RRE migrated to a position coincident with the 4 SL wt RRE. Mutant C RRE migrated slightly faster than Mutant B and the migrations of Mutants D and E were significantly retarded. We next used SHAPE to determine the secondary structure of each RNA, which indicated that they folded as predicted (Figure 2A–E; Supplementary Figures S3–S7). Graphs showing NMIA reactivity plots for all RREs are presented in Supplementary Figure S8. Essentially the same results were obtained when these RNAs were folded by snap cooling and probed with either NMIA or 1M7 (data not shown). Hence, both the non-denaturing gel electrophoresis and SHAPE analysis data supported the predicted secondary structures of the RRE mutants.

### Primary binding of Rev to the RRE and multimerization are largely unaffected by altering SL III/IV secondary structure

**Figure 3.**
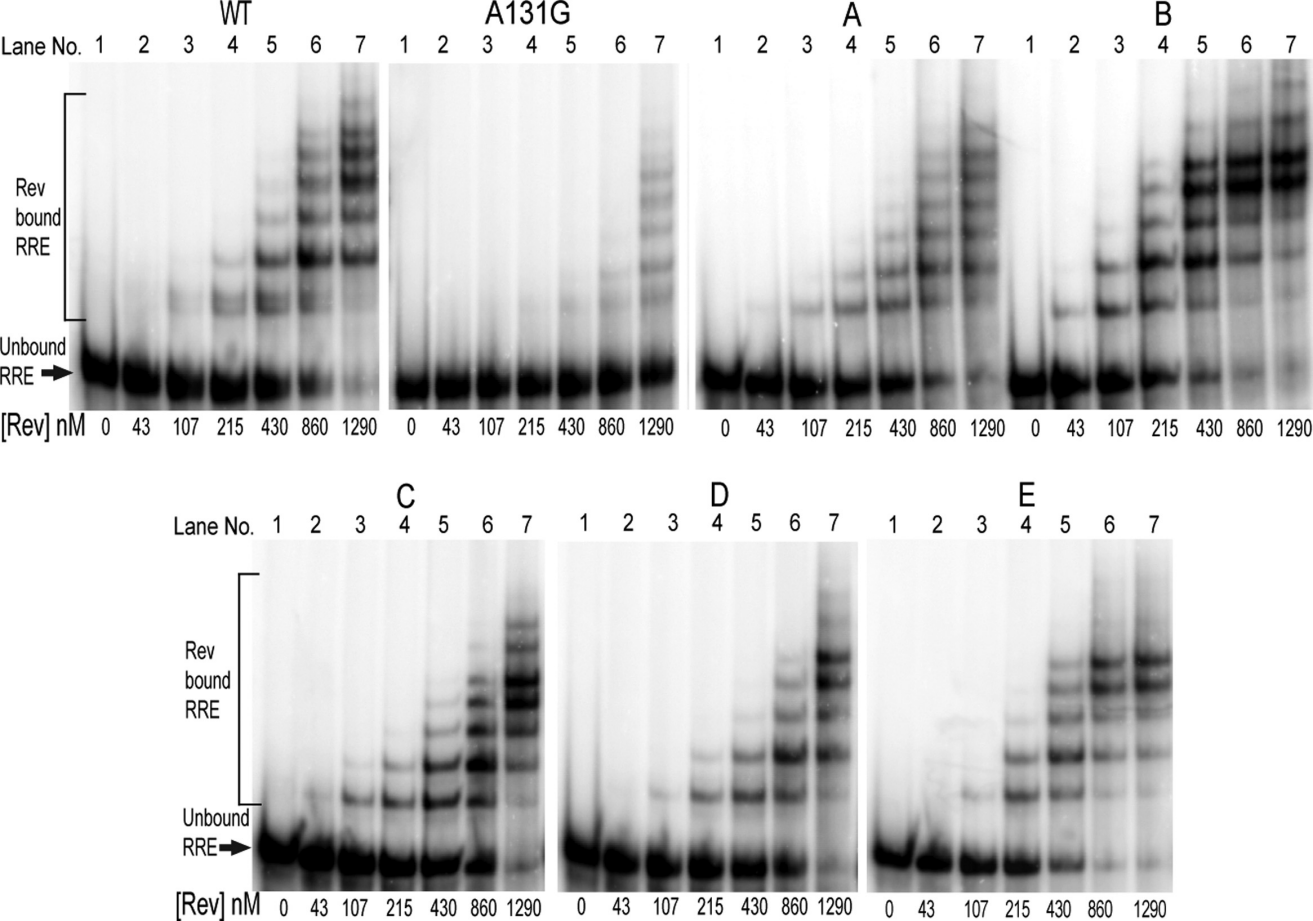
EMSA of wt and mutant RREs. Complexes of Rev and ^32^P-labeled RRE were resolved on a 4% native polyacrylamide gel and detected by phosphorimaging.

We next used an electrophoretic mobility shift assay (EMSA) to test the ability of each RRE mutant to bind Rev *in vitro* and act as a platform for Rev multimerization (Figure 3). In order to establish binding specificity, Rev was incubated with wild-type NL4-3 RRE (wt) or with an RRE containing a mutation in SL IIB (A131G), the primary Rev binding site. Primary binding to the wt RRE, as revealed by the presence of slower migrating complexes, was observed with 107 nM of Rev in the reaction mix. Even slower migrating bands started to appear at 215 nM. In contrast, for the A131G RRE mutant, weak binding was only detected at the highest Rev concentrations.

Having validated the binding assay, we next examined Rev binding to RRE Mutants A–E. Binding assays were repeated with different preparations of Rev and RRE and the results were highly reproducible. Representative examples of the Rev/RRE binding assays are provided in Figure 3. Within the limitations of this gel-based assay, we observed little difference in the concentration of Rev that resulted in the first shifted band, but the pattern of the slower migrating bands appeared different and specific for each mutant. This suggests that the altered RRE configurations in the SL III–IV region had little effect on primary Rev binding but rather affected the stoichiometry and shape of the oligomeric complexes that formed. It is also worth noting that in the wt RRE experiment, doublet bands are clearly evident in the initially shifted RNA bands. Similar doublets are absent in the experiments conducted with the mutant RREs. Thus these results are consistent with our finding that the wt RRE is in fact a mixture of 4 SL and 5 SL configurations and that each of the mutant RREs displays a uniform structure.

### Altering SL III/IV secondary structure affects the ability of the RRE to mediate HIV Gag/Pol expression

**Figure 4.**
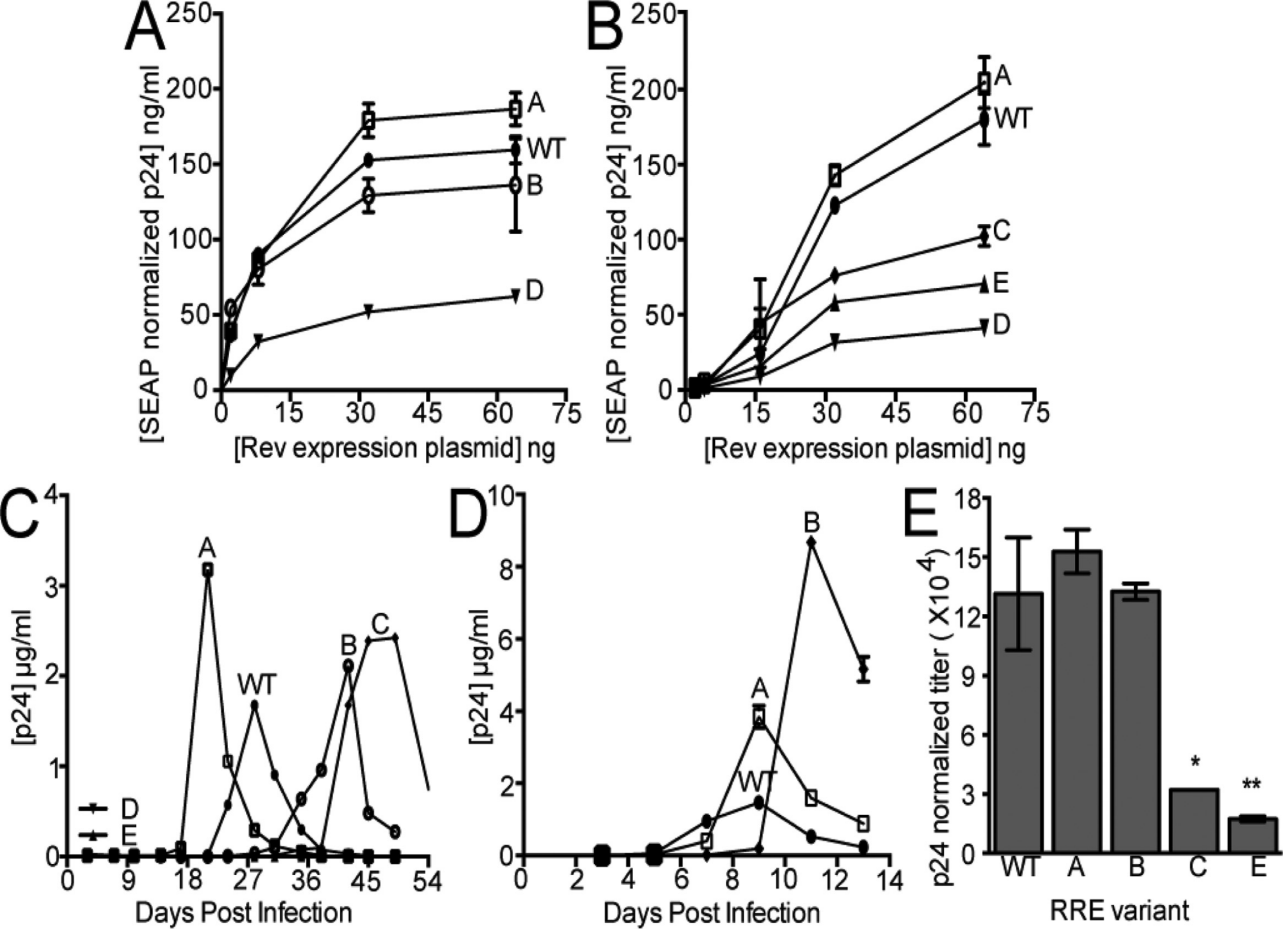
Relative function of wt and mutant RREs. (**A** and **B**) Gag/Pol reporter assay. 293T cells were co-transfected with a GagPol reporter plasmid containing the indicated RREs and increasing amounts of an NL4-3 Rev expression plasmid. A SEAP expression plasmid was used as a normalization control. Medium was assayed 48 h post transfection. Bars show the standard error of the mean from two replicates. (**C**) Spreading infection in SupT1 cells using transfection supernatants. Viral stocks of equal p24, containing the indicated RRE inserted in the nef region, were used to infect SupT1 cells. Each virus also contained an RRE in its normal position, which was inactivated by mutations, that did not change the Env protein sequence. (**D**) Spreading infection of SupT1 cells using titered viral stocks. Supernatants from the cultures in A were used to infect fresh SupT1 cells with an equal MOI. p24 in the medium was assayed as indicated every 3–4 days in C and every 2–3 days in D. (**E**) Assay of vector titer (pTR167nef-) with wt or mutant RREs. Viral vector stocks were prepared for each RRE-containing vector in 293T cells and titered on HeLa cells. *P < 0.05, **P < 0.01, compared to wt.

Each RRE (351 nt) was then tested for activity by inserting it into an HIV-1 GagPol reporter construct previously used to quantitate Rev/RRE function. Each reporter plasmid was transfected into 293T cells together with differing amounts of a plasmid expressing NL4-3 Rev. A third plasmid that expressed secreted alkaline phosphatase (SEAP) was also transfected as an internal control. Under these conditions, Rev/RRE activity could be quantified by measuring the amount of p24 capsid protein secreted into the media and normalizing these values to SEAP expression levels. The results of this experiment (Figure 4A and B) show that each RRE variant promoted a different level of Gag and Gag/Pol expression in response to the concentration of Rev plasmid used. Specifically, the observed activity hierarchy among RRE variants was as follows: Mutant A > wt > Mutant B > Mutants C > Mutant E > Mutant D. These results suggest that the formation of either the SL III/SL IV tandem (5 SL) or a combined SL III/IV (4 SL) confers greater functionality to the RRE than any of the other structural variants. Moreover, the 5 SL arrangement of Mutant A appears to be functionally superior to both the structurally mixed wt RRE and the exclusive 4 SL configuration of Mutant B.

### Altering RRE SL III/IV secondary structure affects HIV replication rates in a spreading infection

To determine how variations in RRE secondary structure affect RRE function in the context of virus replication, we tested the ability of each mutant RRE to promote HIV replication in the context of a spreading infection in SupT1 cells. Normally, mutations in the RRE would be expected to alter or disrupt the envelope protein coding region, making a comparison of the replication rates of viruses with different RRE mutations problematic. To overcome this problem, we inserted each 351 nt long RRE variant into the Nef region of an NL4-3 proviral clone in which the native RRE was inactivated by mutations that did not change the envelope protein sequence (58). Since Nef is not required for HIV replication in SupT1 cells, this allowed us to create a series of replication competent proviral clones that were isogenic except for the mutated RRE under examination. Proviral clones were transfected into 293T cells and all produced sufficient p24 in these cells for generation of viral stocks.

SupT1 cells were then infected with transfection supernatants containing equal amounts of p24. Cells were maintained in long-term culture by replacing 60% of the culture every 3rd–4th day with fresh medium, and virus replication was monitored by measuring the amount of p24 released into the supernatant. This experiment showed that each mutant virus replicated at a different rate, with the following order of efficiency: Mutant A > wt > Mutant B > Mutant C (Figure 4C). No detectable replication was observed for Mutants D and E. To ensure that these observations were not due to variations of p24 to infectivity ratios of the transfection supernatants, SupT1 supernatant fractions from the wt, Mutant A and Mutant B infections shown in Figure 4C were harvested and used to infect fresh SupT1 cells, normalizing input by MOI as determined using a TCID_50_ assay (Figure 4D). Although replication times were compressed, the relative replication kinetics of passaged viral variants were essentially the same as in the previous experiment (cf. Figure 4D and C). Thus, in accordance with the data obtained using the p24 reporter assay, these data indicate that the secondary structure assumed by RRE nucleotides 163–221 is important for RRE function during virus replication, and that either a tandem SL III/SL IV or a combined SL III/IV structure is preferred. In addition, the data show that the 5 SL RRE variant promotes HIV replication in SupT1 cells more efficiently than either the wt or the 4 SL variant.

### Abrogation of the SLIII/IV secondary structures in the native RRE diminishes the titer of a Rev-dependent HIV vector

To test each of the mutant RREs in their natural context within the envelope coding region, we utilized a modified proviral vector (pTR167nef^−^) (59) (see Supplementary Figure S9). This vector was created by deleting 5491 nt from the central region of pNL4-3 and inserting an SV40 early promoter-driven hygromycin resistance gene (Hyg^r^) in the middle of *env*. It also has a small deletion that inactivates Nef function. When co-transfected with an HIV packaging system, the vector produces a modified HIV genomic RNA that contains a complete intron and whose trafficking is dependent on Rev–RRE function. This genomic RNA is also packaged into virus-like particles that are capable of transducing hygromycin resistance to target cells. In the target cell, the mRNA encoding hygromycin resistance is not Rev-dependent, since it does not contain a complete intron. Thus the magnitude of the virus titer derived from the producer cell is directly reflective of Rev–RRE activity in those cells.

Mutant RREs were exchanged with the wt NL4-3 counterpart to create a series of isogenic vectors differing only in their RRE mutations. In this context, each 351 nt long RRE is in its normal position within the *env* intron and is surrounded by about 450 nt upstream and 550 nt downstream of authentic HIV sequence. Vectors were transfected into 293T cells together with plasmids that supplied Gag/GagPol, Tat, Rev and VSV-G. Supernatants were collected after 48 h, assayed by p24 ELISA and titered for hygromycin-resistant colony formation on HeLa cells. Figure 4E clearly shows that the titers of the viruses containing mutant RREs that could not assume either the normal 4 SL or 5 SL structure (i.e. Mutants C and E) were greatly reduced relative to the titers of the viral vectors containing wt, Mutant A or Mutant B RREs. Moreover, although the differences in titer among wt, Mutants A and B viruses did not reach statistical significance, the titer of Mutant A trended slightly higher than those of the other two variants. Taken together, these data indicate that the SL III/IV region, which lies outside the primary Rev binding site, must assume either the 4 SL or 5 SL structure for correct RNA trafficking and packaging into virus particles. However, this experiment failed to show a clear difference in activity between wt RRE and the stable 4 SL and 5 SL mutants.

### Competition growth experiments demonstrate a replicative advantage for virus containing a 5 SL RRE

The data presented thus far suggest that the 5 SL RRE is more active than the 4 SL or mixed (wt) structures. However, since the differences we observed were slight, we compared these mutants and a virus containing the wt RRE in direct competition growth assays. To perform these assays, two viruses, each with a different RRE inserted into the Nef region, were added to the same culture of SupT1 cells at an equal MOI of 0.000005. Viruses were then allowed to replicate and spread throughout the cultures for 12 days, at which time the cultures were harvested and cellular DNA was recovered. The relative amounts of integrated proviral DNA produced by each virus was measured using a polymerase chain reaction (PCR)-based heteroduplex tracking assay (HTA). To perform this assay, PCR was performed on each DNA sample using primers spanning the SL III/IV regions of the RRE. The resulting amplicons were then denatured and hybridized to ^32^P-labeled amplicons containing the wt sequence from the same region and the products formed were fractionated by non-denaturing gel electrophoresis. Since sequence mismatches within the wt/Mutant A and wt/Mutant B heteroduplexes caused them to migrate differently from the perfectly base-paired wt/wt homoduplex, as well as from each other, they were easily separated by native gel electrophoresis and quantified by phosphorimaging (Supplementary Figure S10).

**Figure 5.**
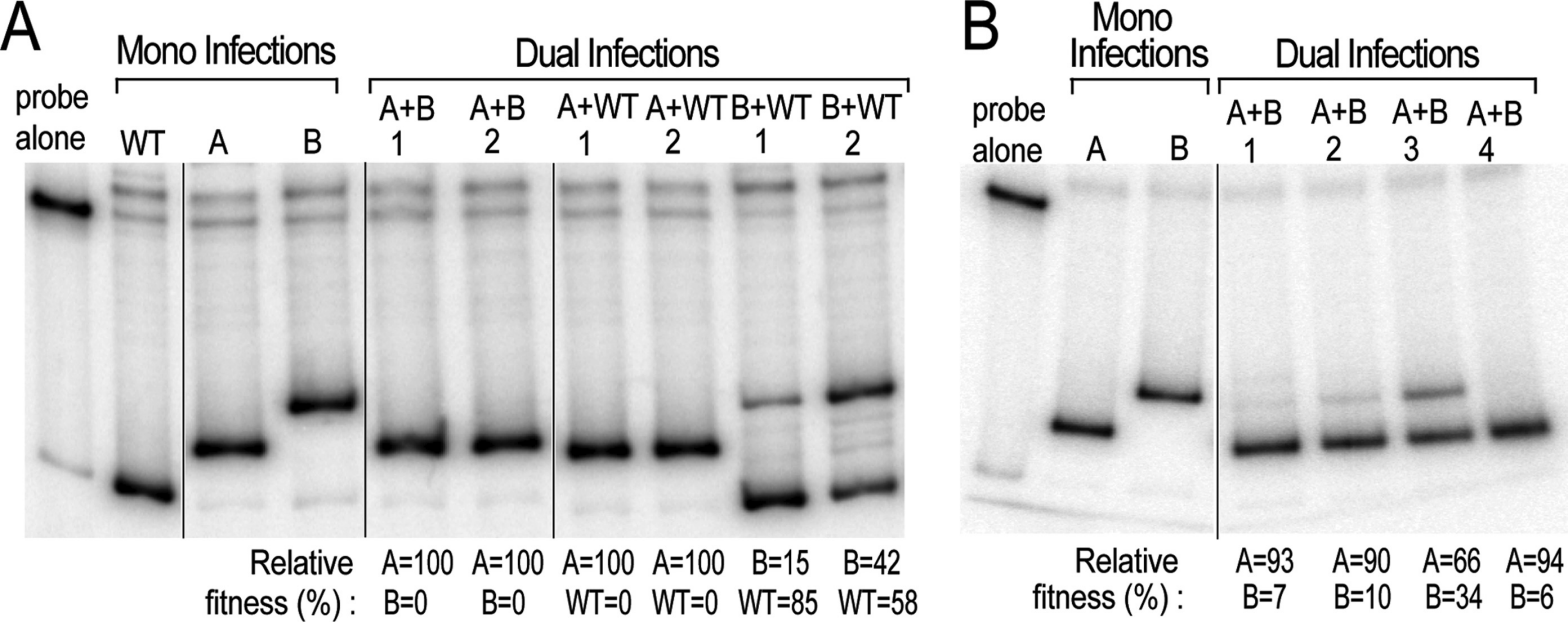
Competition assays using viruses with wt or mutant RREs. **A** and **B** represent two independent experiments. SupT1 cells were either singly or dually infected as indicated. DNA was extracted 10 days post infection, PCR performed and the amount of each viral sequence present was analyzed by the HTA, using ^32^P-end labeled wt RRE probe run on an 8% native polyacrylamide gel. The relative percent contribution of each species in the DNA extracted after 10 days is shown below each panel.

Figure 5A and B shows the results of two different experiments. Lanes on the left side of each panel show bands generated from mono infections of the wt, Mutant A and Mutant B viruses, thereby establishing the migration position of each species. Bands derived from the respective virus variants in the dual infections were then quantified and used to calculate a relative replicative ‘fitness’ value, similarly to procedures used to determine replicative fitness of viruses from different HIV clades (56). The results provide strong evidence that the 5 SL RRE conveyed a selective growth advantage over both the wt and 4 SL variant and that the wt, mixed-conformation RRE conveyed a selective growth advantage over the 4 SL variant.

## DISCUSSION

In this study, we re-examined the secondary structure of the HIV-1 RRE and showed that the wt NL4-3 RRE exists in an approximately equimolar mixture of 4 SL and 5 SL conformations. This finding is consistent with a previously published study reporting that the wt NL4-3 RRE migrated as a doublet on native polyacrylamide gels (60). In that study, the authors speculated that the two bands might correspond to the 4 SL and 5 SL structures. However, attempts to demonstrate a difference in conformation of the RNA in each of the two bands by atomic force microscopy were unsuccessful. The application of in-gel SHAPE to this issue has now allowed us to demonstrate that the HIV-1 RRE can indeed assume two distinct conformations.

By creating mutants capable of forming only one or the other conformer, we demonstrated functional differences between the two structures and showed that the 5 SL structure was more active in promoting viral replication than its 4 SL counterpart. These replication results cannot be easily explained by either differences in primary RRE–Rev binding affinity or in Rev binding cooperativity, since our electrophoretic mobility gel shift experiments failed to show consistent quantitative differences between wt and any of the RRE mutants. However, a close inspection of the gels reveals slight differences in the relative migration rates and relative stoichiometry of the 4 SL and 5 SL complexes, suggesting differences in the conformation of the Rev oligomer formed. These differences could translate into different levels of function, by changing the efficiency of how each complex recruits the additional proteins needed for Rev to function *in vivo*. Two recent papers provide some insight into the mechanisms that could generate complexes with different Rev oligomer conformations. In the first paper, the authors solved the crystal structure of a Rev dimer complexed to a fragment of the RRE that contained the primary Rev binding site and compared it to the structure of the dimer alone in solution. It was found that Rev–RRE binding caused a dramatic change in the conformation of the Rev dimer, due to a considerable structural flexibility within its dimerization domain (61). In the second paper, the authors used single particle electron microscopy to visualize the interaction of the Rev/RRE complex with Ran-GTP and the export receptor Crm1. They conclude that the Rev-Ran-GTP-RRE complex recruits Crm1 and causes it to dimerize by a mechanism that is dependent on the arrangement of Rev nuclear export signals in the Rev oligomer (62). While these studies suggest that alterations in RRE RNA conformation may lead to differently organized Rev/RRE complexes, which support different levels of functional activity, a detailed understanding of how the 4 SL and 5 SL RRE promote different rates of viral replication will require further structural studies, including both *in vivo* and *in vitro* structural determinations. Nevertheless, our results clearly show that the RRE is a dynamic structure that can serve to modulate and fine tune the rate of HIV replication *in vivo*.

A detailed three-dimensional structure of the RRE, as determined by small angle X-ray scattering (SAXS), was recently reported (63). By modeling the 4 SL secondary structure of the RRE on the SAXS envelope, the authors deduced a SAXS structural model. However, the study did not attempt to fit the 5 SL RRE structure into the SAXS envelope; thus, it sheds no light on the basis for the functional differences observed here. Another recent *in vitro* study examined the structure of the 354-nt ARV/SF-2 RRE using SHAPE and SAXS with or without added Rev protein (49). The authors found that the center of SL I in the long unbound RRE made long-range contact with a central area of the RRE that included regions of the central loop, SL IV and SL V. In time-resolved SHAPE experiments performed in the presence of Rev, they first observed changes in SHAPE reactivities in SL IIB, which were immediately followed by reactivity changes in the three way junction of SL IIA, SL IIB and SL IIC. At a much later time point, SHAPE reactivity changes were also observed in the center of SL I. Based on this observation, the authors propose a model where the Rev initially binds rapidly to SL IIB and the three-way junction. This initial Rev binding is then proposed to expose a cyptic Rev binding site at the center of SL I, leading to the formation of the final Rev/RRE RNP. While it has not yet been determined if either the NL4-3 RRE or our functionally active Mutant A and B RREs interact with Rev in this manner, it is tempting to speculate that the differential activities observed may be a reflection of how efficiently they are able to carry out this rearrangement once Rev is bound. Further experimentation will be required to address this issue.

Several studies have indicated potential links between HIV Rev/RRE function and pathogenesis. For example, Bobbitt *et al*. (64) found that in asymptomatic infected patients with an active immune response, infected cells were more resistant to anti-Gag and anti-Env cytotoxic T-lymphocyte killing compared to infected cells from advanced AIDS patients. This was attributed to lower expression levels of Gag and Env in cells of the healthy patients, resulting from attenuated Rev alleles. Other studies have also reported the contribution of less-active *rev* alleles to long-term survival of HIV-1 infection in some patients (65,66) and there have also been a few studies on the evolution of Rev and RRE function in individual patients. These studies have concluded that RREs of varying activity can evolve over time (67,68). RRE functional variation has also been studied in a longitudinal cohort (1,68,69), where a correlation was observed between the rates of CD4+ decline and RRE activity at the late time points. Thus, RRE evolution may be an important regulator of HIV pathogenesis and disease progression.

In the present study, we have shown that HIV can achieve differential replication rates simply by alternating RRE structure, which presumably might occur in the absence of virus evolution. The ability of the RRE to adopt different structural conformations that promote different replication activities may allow HIV to modulate its rate of replication under distinct conditions, or in distinct compartments, to better survive in the environment of the host. Another example where HIV RNA has been shown to form two alternative RNA structures is in a region within the HIV-1 RNA 5′ leader (70,71). In one of the structures formed, there is base-pairing between the poly A addition site and the dimer initiation site (DIS) which blocks RNA packaging and allows the Gag start codon to remain single stranded. This enables the RNA to form polyribosomes for the translation of Gag. In the second structure, the Gag start codon is found base paired, but the DIS is not, and thus the RNA is free to promote dimerization and the packaging of genomic viral mRNA. Thus, the particular structure that the RNA adopts regulates whether the RNA is translated or packaged. Studies with other RNA viruses have also revealed many examples of regulation that is mediated by alternative RNA structures (72–74).

It is striking that HIV has evolved an RNA export element that is larger and more complex than the CTEs found in the simpler viruses and it has remained a puzzle as to why such a large RNA element has been conserved. It may be that the complex structure of the RRE is necessary to confer the ability to rearrange and promote differential Rev activity. This might provide a novel heretofore unstudied mechanism to regulate virus growth in response to various cellular cues. While factors promoting formation of different RRE conformers remain to be elucidated, it is striking that the free energies of each are extremely similar, making it plausible that minor differences in the concentration of yet unidentified cellular factors could dynamically influence RRE folding *in vivo*.

## SUPPLEMENTARY DATA

Supplementary Data are available at NAR Online.

SUPPLEMENTARY DATA
