# Supplementary material for: The HIV-1 Rev response element (RRE) adopts alternative conformations that promote different rates of virus replication
Source: Nucleic Acids Res. 2015 Apr 8;43(9):4676–86. doi: 10.1093/nar/gkv313 (PMC4482075; doi:10.1093/nar/gkv313)
Supplement: SUPPLEMENTARY DATA [file supp_43_9_4676__index.html]

The HIV-1 Rev response element (RRE) adopts alternative conformations that promote different rates of virus replication — The HIV-1 Rev response element (RRE) adopts alternative conformations that promote different rates of virus replication — SUPPLEMENTARY DATA 

# The HIV-1 Rev response element (RRE) adopts alternative conformations that promote different rates of virus replication

## SUPPLEMENTARY DATA

**Files in this Data Supplement:**

- SUPPLEMENTARY DATA
