# Supplementary material for: The HIV-1 Rev response element (RRE) adopts alternative conformations that promote different rates of virus replication
Source: Nucleic Acids Res. 2015 Apr 8;43(9):4676–86. doi: 10.1093/nar/gkv313 (PMC4482075; doi:10.1093/nar/gkv313)
Supplement: SUPPLEMENTARY DATA [file supp_gkv313_nar-03626-r-2014-File007.pdf]

Supplemental Data

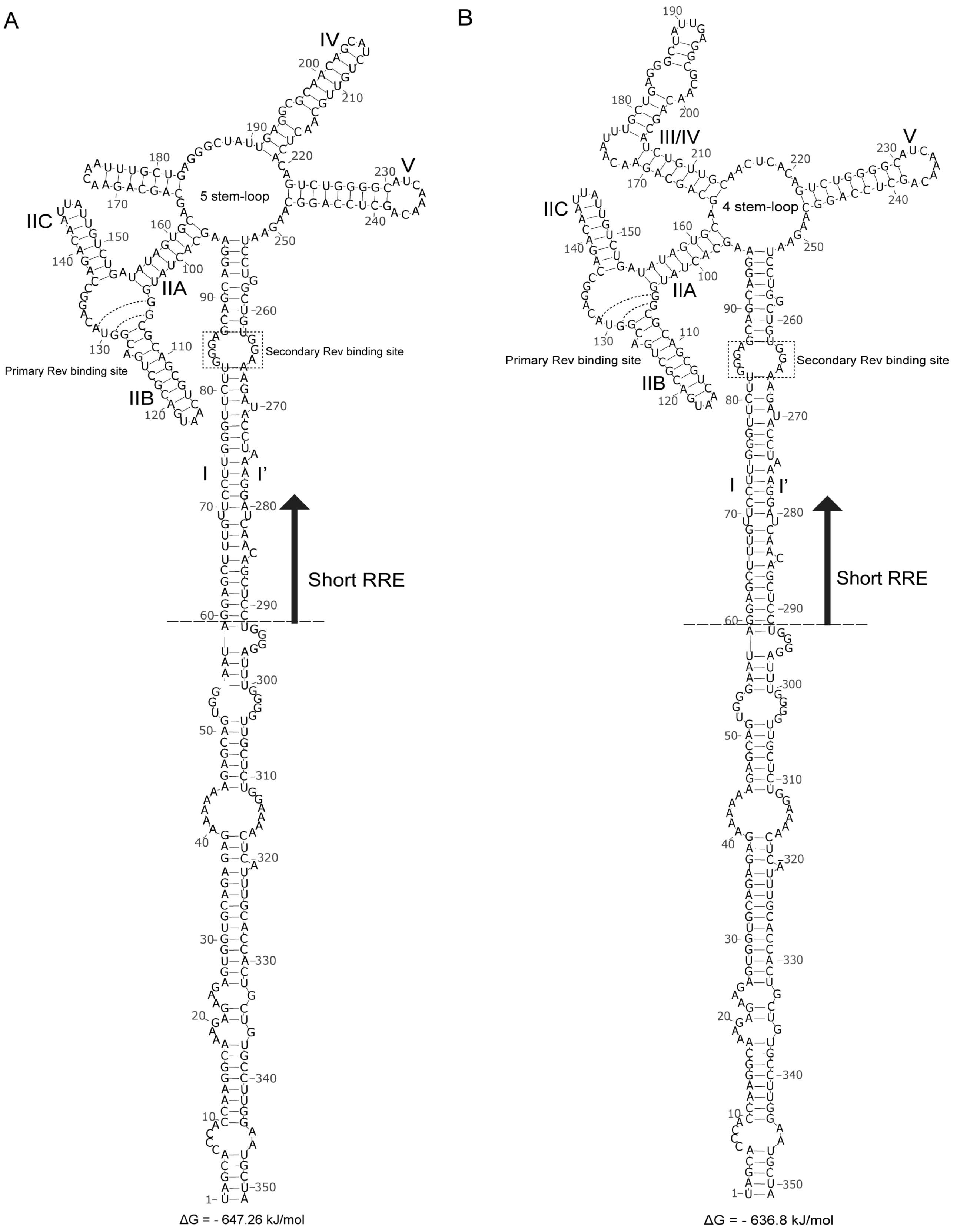

Fig. S1: The two secondary structures of 351 nt (long) RRE. A. The 5 stem-loop structure of long RRE. B. The 4 stem-loop structure of long RRE. The 351nt long RRE structure is defined by nucleotides 7701-8050 of pNL4-3 (Gen-Bank accession no. AF324493). The composition of the short RRE (232 nt) and the primary and secondary Rev binding sites are indicated.

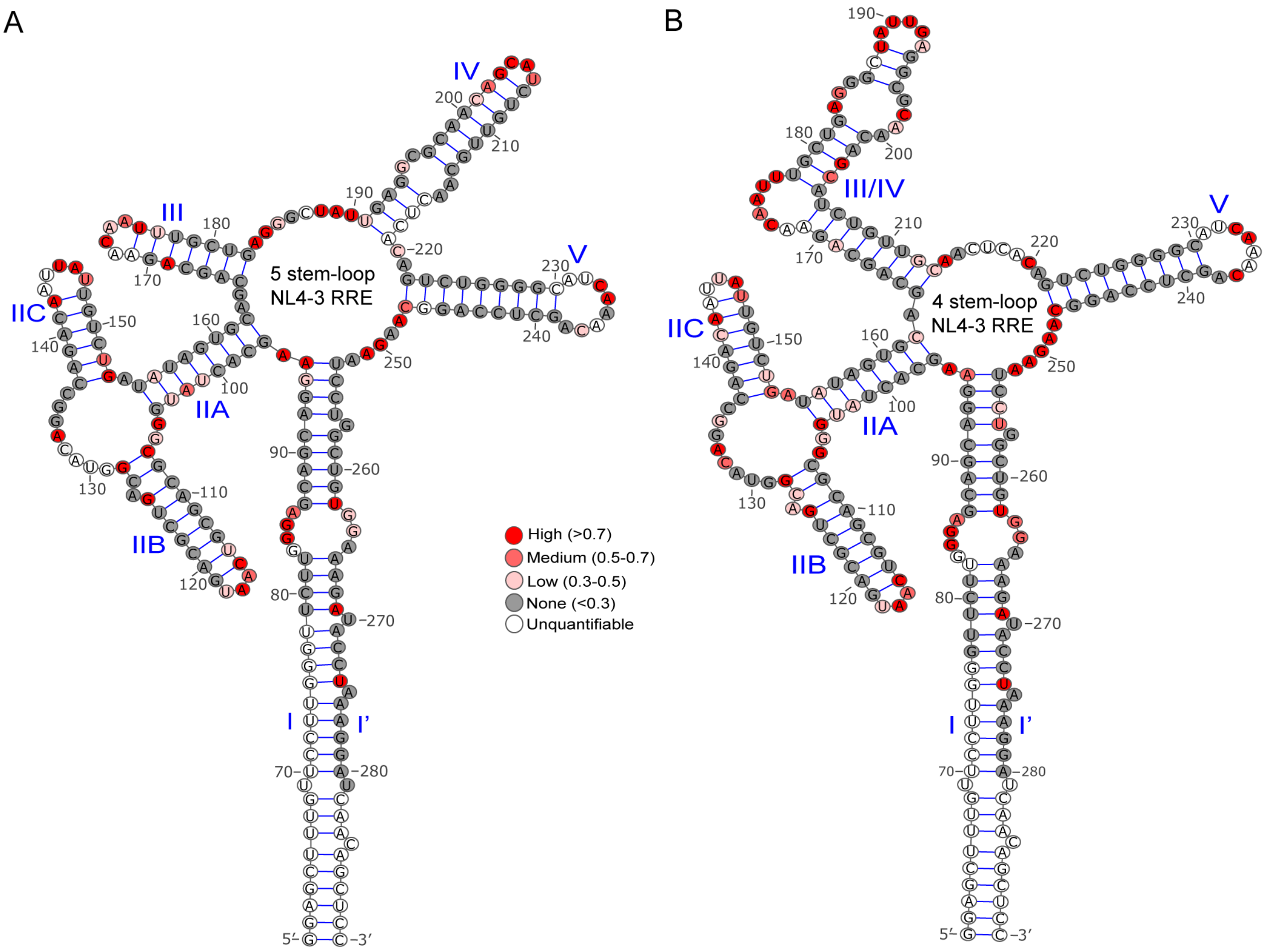

Fig. S2: The two secondary structures of the 232 nt RRE determined by in-gel SHAPE. A. The 5 stem-loop RRE structure is the energetically most favorable RRE structure in the faster migrating RRE band. B. The 4 stem-loop RRE structure is the energetically most favorable RRE structure in the slower migrating RRE band. In-vitro transcribed WT RRE RNA, folded by slow cooling at 25°C, was resolved into two bands on an 8% native polyacrylamide gel. RRE within each excised gel band was probed with NMIA and further processed as described in Methods. Secondary structure was generated by RNAstructure 5.5 software using experimentally determined SHAPE reactivity constraints applied according to the default slope (1.8kcal/mol) and intercept (-0.6kcal/mol) in the pseudo-energy equation.

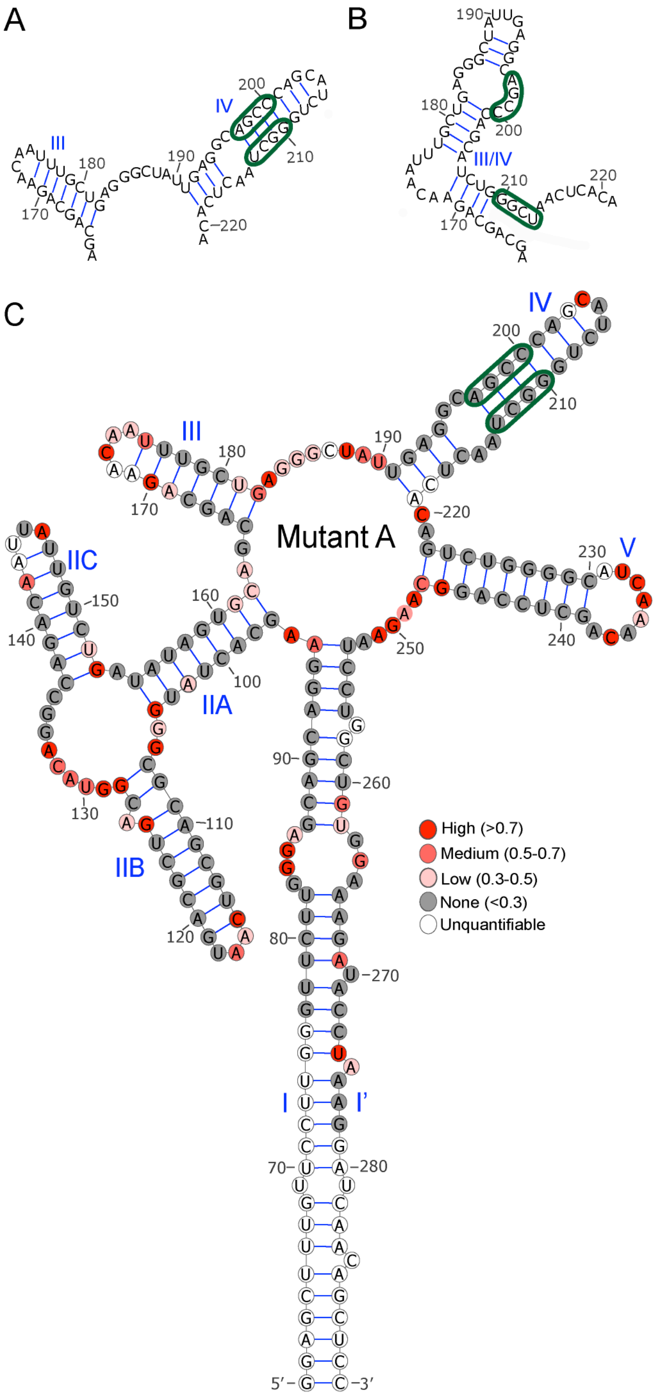

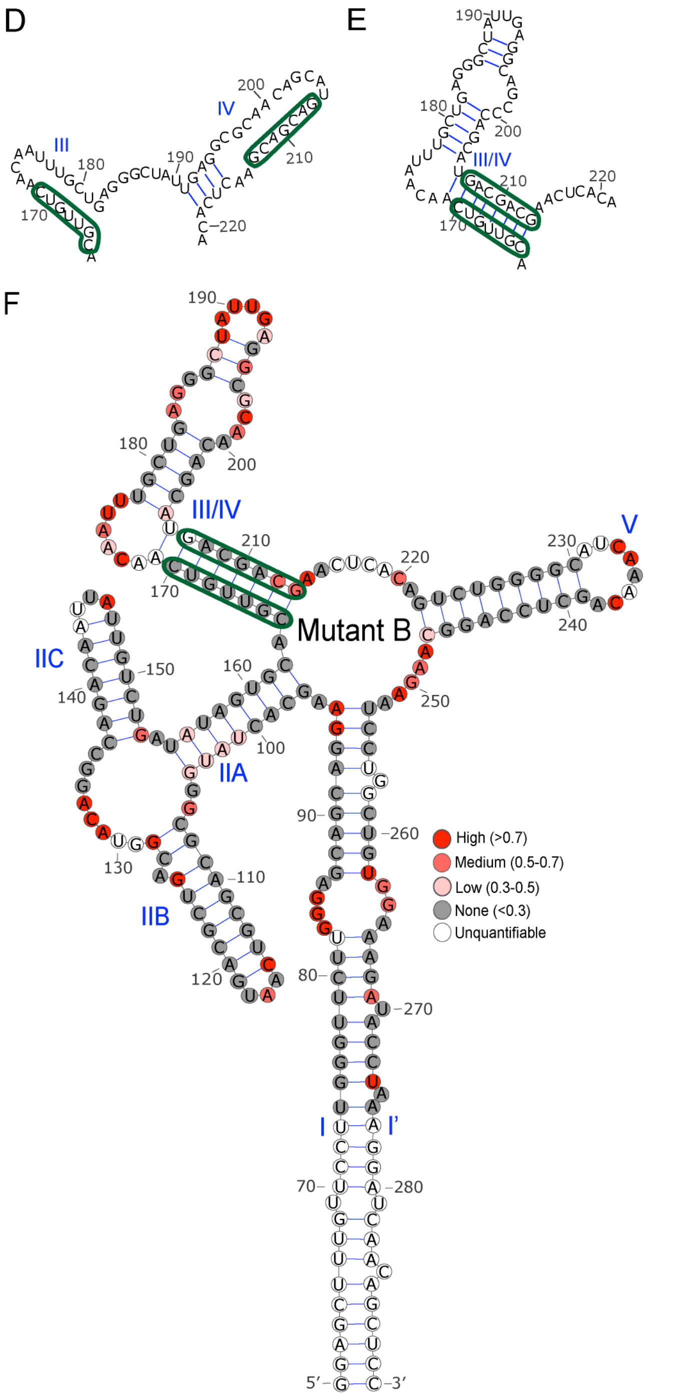

Fig. S3: Predicted and SHAPE-derived secondary structures of the Mutant A RRE variant. A. and B.: Nucleotide substitutions used to generate Mutant A mapped on the 5 and 4 stem-loop WT RRE structures, respectively (only the stem-loop III and stem-loop IV regions are shown). C. SHAPE-derived secondary structures of RRE mutant A. Mutated nucleotides are enclosed by the green border. The structure was determined as described in Methods and Fig. S2.

Fig. S4: Predicted and SHAPE-derived secondary structures of the Mutant B RRE variant. A and B.: Nucleotide substitutions used to generate Mutant B mapped on the 5 and 4 stem-loop WT RRE structures, respectively (only the stem-loop III and stem-loop IV regions are shown). C. SHAPE derived secondary structures of mutant B. The mutated nucleotides are enclosed by the green border. The structure was determined as described Methods and Fig. S2..

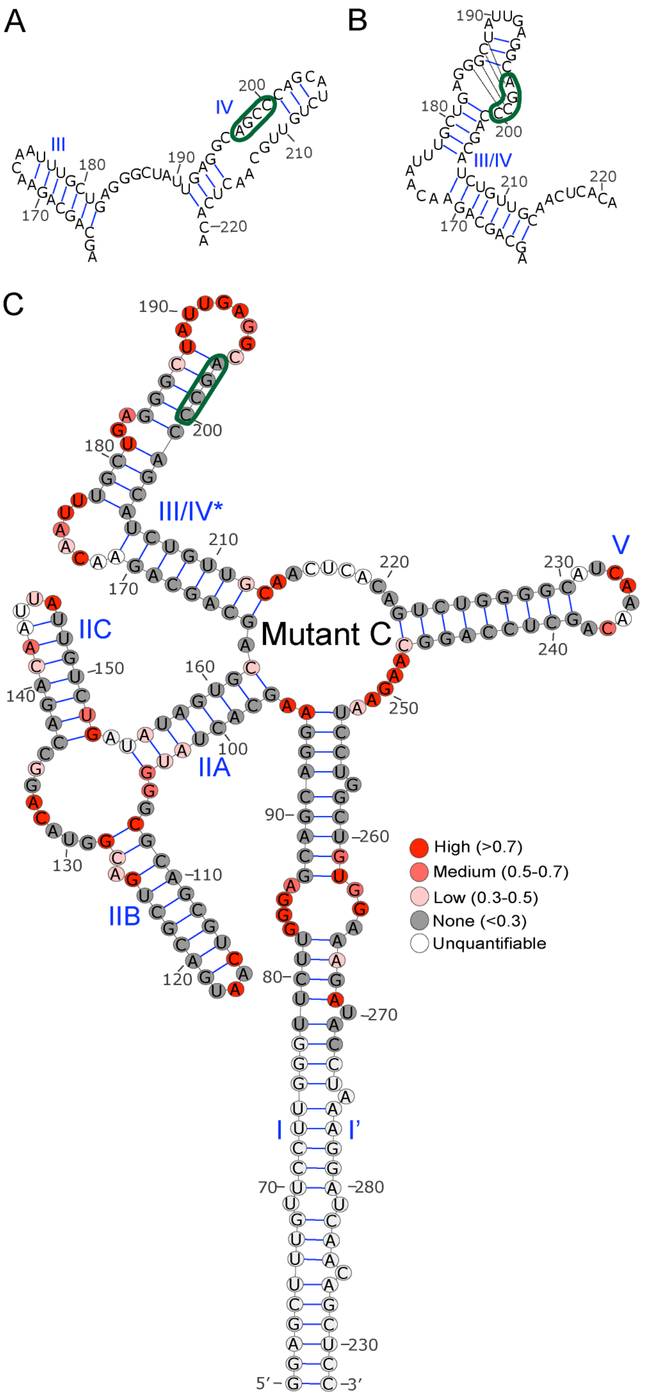

Fig. S5: Predicted and SHAPE-derived secondary structures of the Mutant C RRE variant.. A and B.: Nucleotide substitutions used to generate Mutant C mapped on the 5 and 4 stem-loop WT RRE structures, respectively (only the stem-loop III and stem-loop IV regions are shown).The potential complementarity of the introduced mutations to other bases in SL III/IV is shown by dashed lines. C. SHAPE derived secondary structures of mutant C. The mutated nucleotides are enclosed by the green border. The structure was determined as described Methods and Fig. S2.

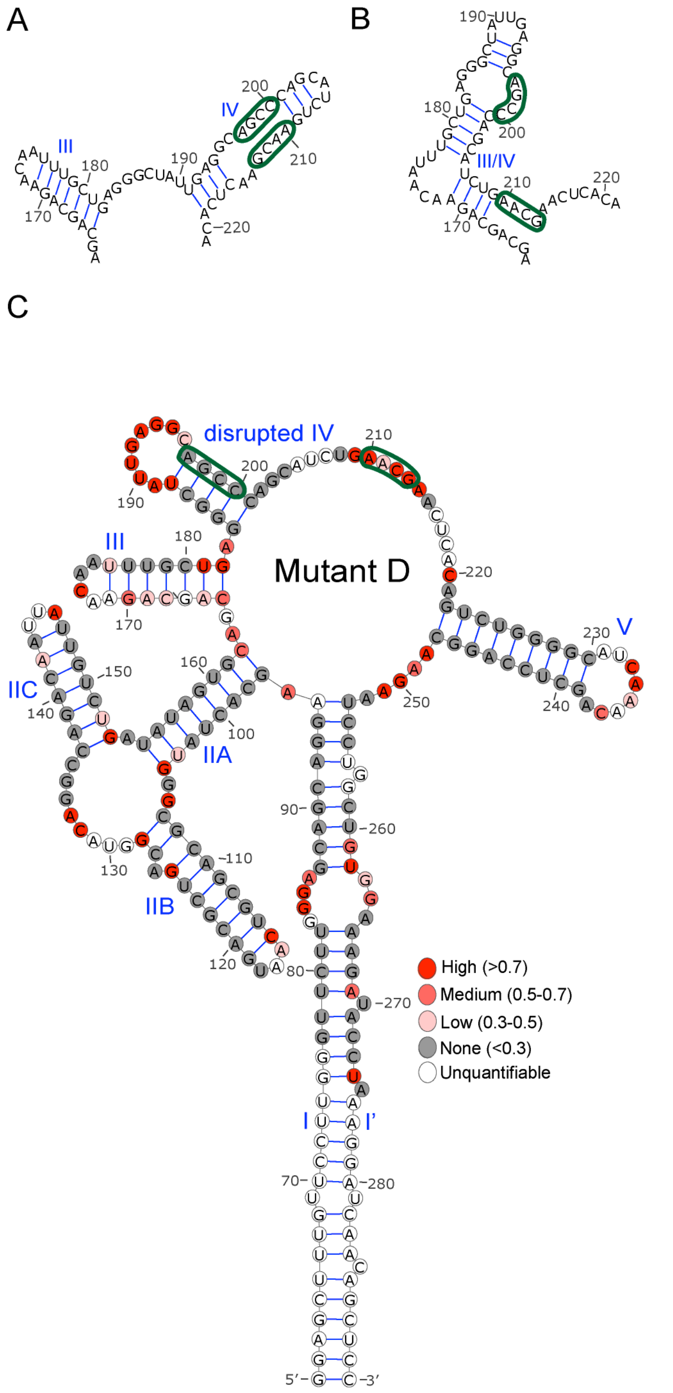

Fig. S6: Predicted and SHAPE-derived secondary structures of the Mutant D RRE variant.. A and B.: Nucleotide substitutions used to generate Mutant D mapped on the 5 and 4 stem-loop WT RRE structures, respectively (only the stem-loop III and stem-loop IV regions are shown). C. SHAPE derived secondary structures of RRE mutant D. The mutated nucleotides are enclosed by the green border. The structures were determined as described in Methods and Fig. S2.

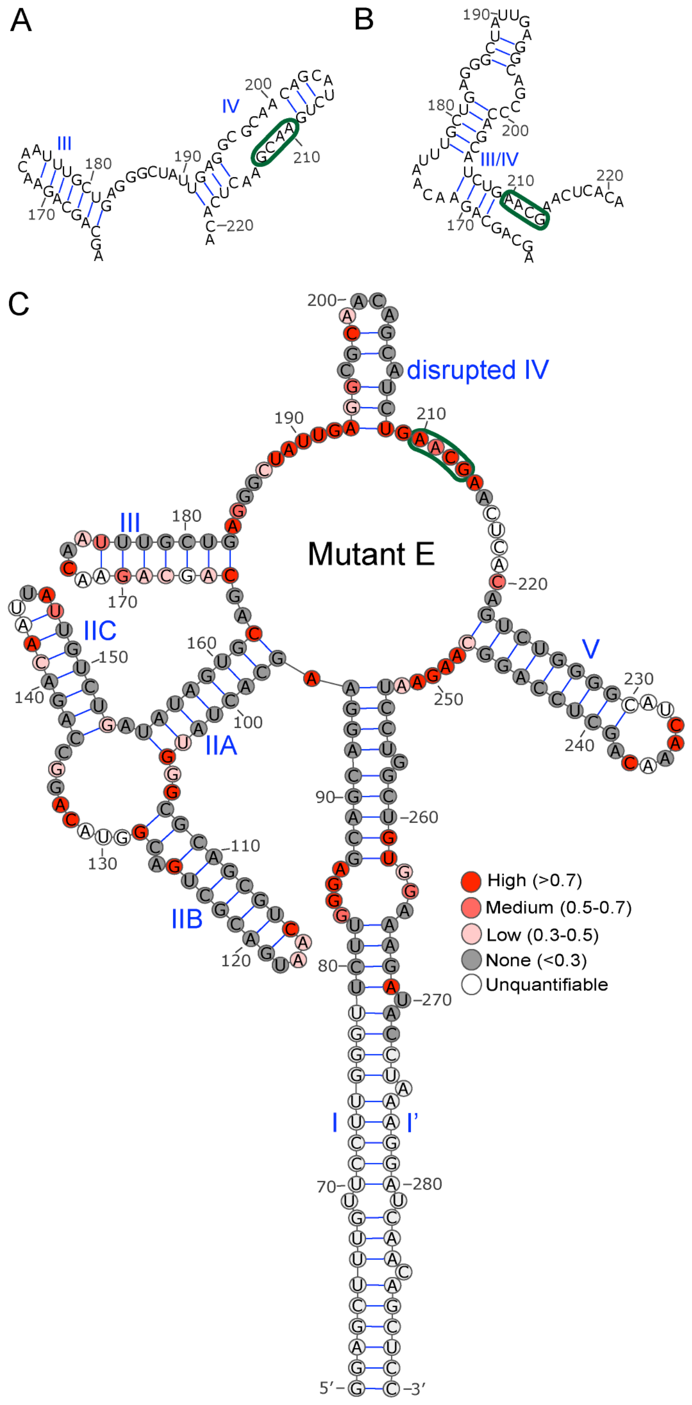

Fig S7: Predicted and SHAPE-derived secondary structures of the Mutant E RRE variant.. A and B.: Nucleotide substitutions used to generate Mutant E mapped on the 5 and 4 stem-loop WT RRE structures, respectively (only the stem-loop III and stem-loop IV regions are shown). C. SHAPE derived secondary structures of RRE mutant E. The mutated nucleotides are enclosed by the green border. The structures were determined as described in Methods and Fig. S2.

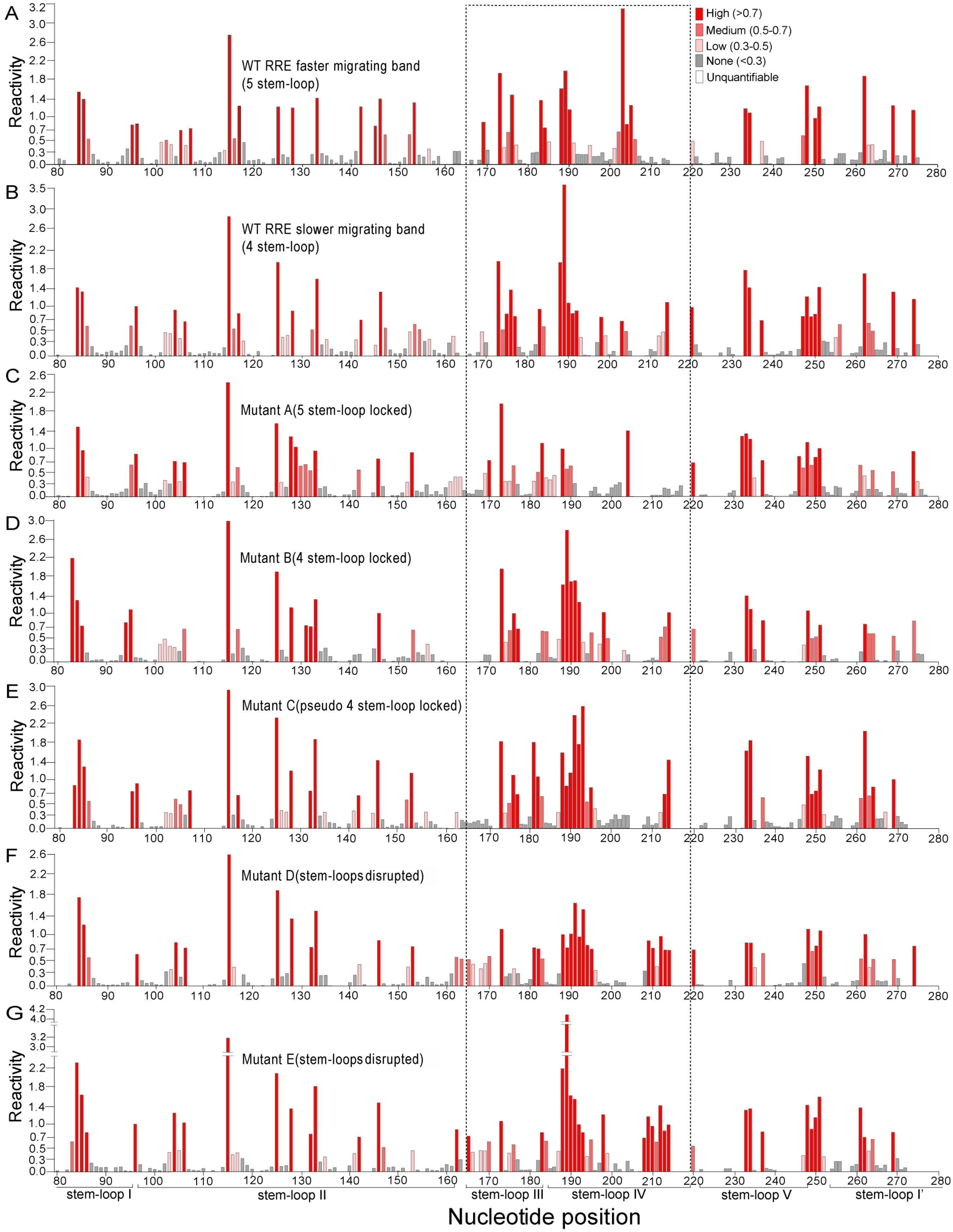

Fig. S8: Comparison of the SHAPE reactivity plots of the two structural forms of the WT RRE derived by in-gel SHAPE and of the mutant RREs derived by conventional SHAPE.

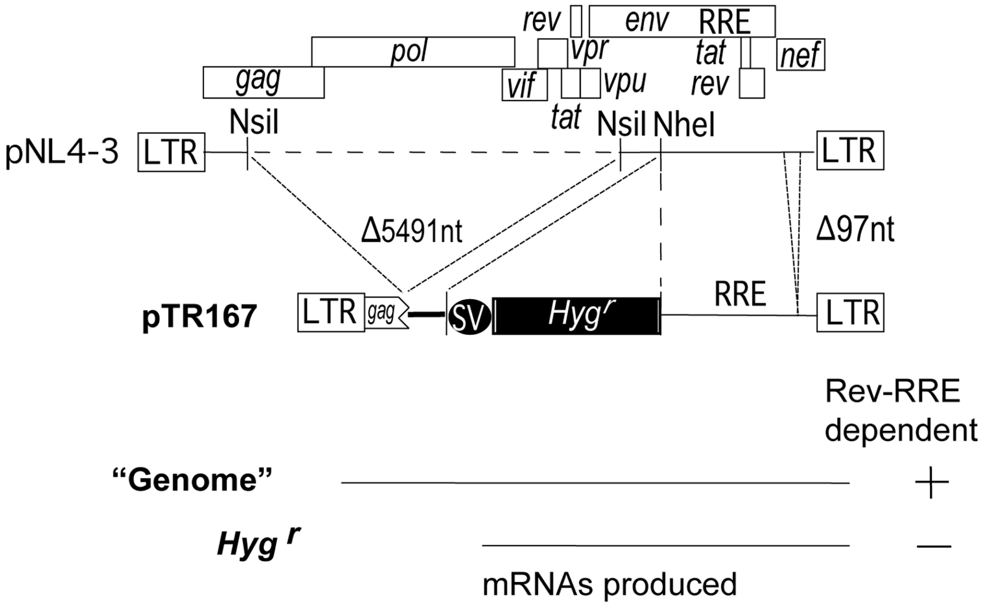

Fig. S9: Schematic representation of pTR167 nef- construct. The construct was derived from pNL4-3 as shown and contains a cassette with the hygromycin resistant gene (hygr) driven by an SV40 early promoter. Each test RRE was cloned into the native RRE position. The full length genomic RNA contains a complete intron and thus is dependent of Rev for nucleocytoplasmic export. The mRNA encoding hygromycin resistance does not contain a complete intron and is Rev-independent.

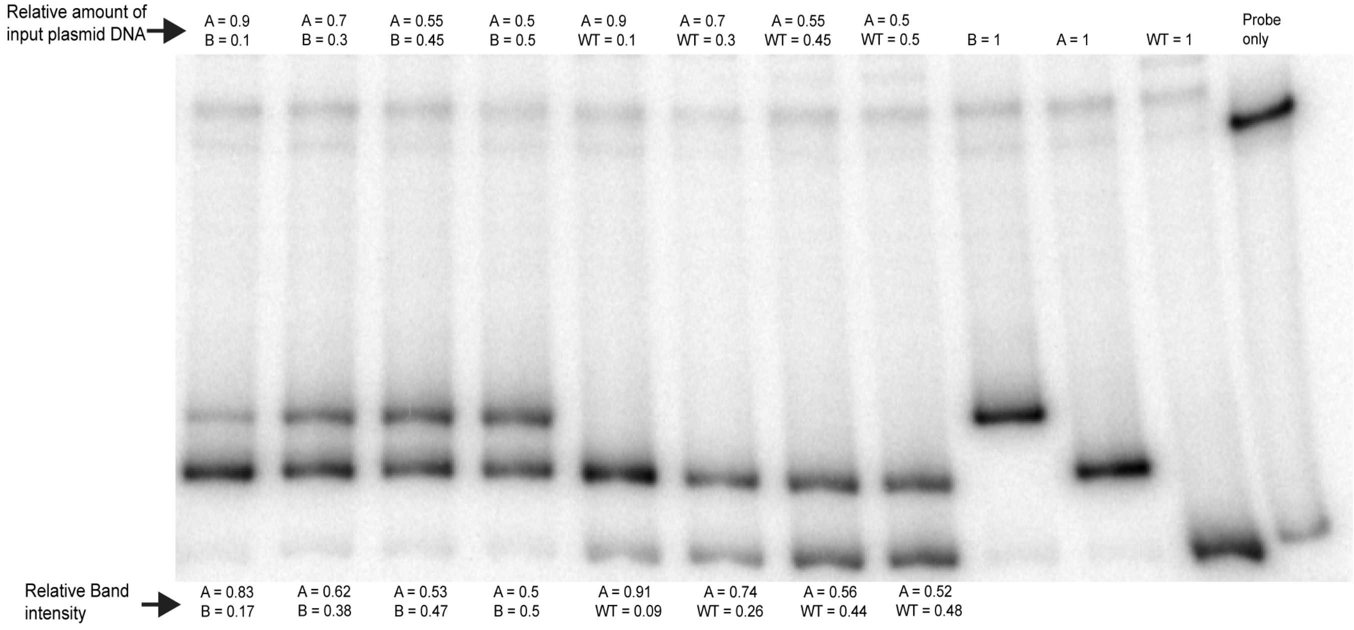

Fig. S10: Control HTA Demonstrating Quantitation. Different ratios and combinations of WT, mutant A, and mutant B RRE containing proviral plasmids were amplified by PCR using a specific primer pair. The PCR product was then melted and re-annealed to γ32P-end labeled WT RRE probe that was also generated by PCR. The mixtures were analyzed on an 8% native polyacrylamide gel run at room temperature for 3 hours and quantified by phosphorimaging.

Extended Materials and Methods:

HIV Proviral Constructs, Expression Plasmids and HIV Vectors: All the constructs used in the study are referenced by their Hammarskjöld-Rekosh laboratory archive number with a prefix pHR. Additional details about their construction are available upon request.

Infectious proviral clones contained a 393-nt RRE region corresponding to nt 7676 to 8068 in pNL4-3 inserted into the Nef region. This region included 24 nt upstream and 17 nt downstream of the 351 nt RRE. The proviruses also contained a non-functional RRE generated by multiple third base mutations with the RRE which did not change the coding sequence of the overlapping env (1) and a 97 bp deletion that inactivated the Nef gene. The plasmids with wt RRE, mutant A, mutant B, mutant C, mutant D, and mutant E inserted into Nef were archived as pHR 4874, pHR 4884, pHR 5121, pHR 4876, pHR 4880, and pHR 4878 respectively.

The GagPol reporter plasmids used in this assay all had the 393 nt sequence containing the 351 nt RRE and flanking regions inserted immediately after the GagPol coding region in the vector pCMV-GagPol-RRE in place of the original RRE (2). The plasmids with the wt RRE, mutant A, mutant B, mutant C, mutant D, and mutant E were archived as pHR 4824, pHR 4908, pHR 5119, pHR 4808, pHR 4812, and pHR 4810 respectively.

The HIV vector pTR167 (3) was modified to contain the wt and mutated RREs and the 97 bp deletion that inactivated Nef. The plasmids with the wt RRE, mutant A, mutant B, mutant C, mutant D, and mutant E were archived as pHR4910, pHR5054, pHR4406, pHR4438 and pHR4408 respectively.

Each mutant RRE was amplified from one of the appropriate plasmids described above and inserted into the pCR^{TM}2.1-TOPO vector (Invitrogen) for RNA transcription as described below. The plasmids with the wt RRE, mutant A, mutant B, mutant C, mutant D, and mutant E were archived as pHR4854, pHR5083, pHR5091, pHR5081, pHR5089 and pHR5085 respectively.

pCMV (pHR16), pCMVSEAP (pHR1831), pCMV VSV-G (pHR2004) have been previously described (2).. pCMVRev-NL4-3 (pHR3821) was modified from pCMVRev (pHR16) to contain a codon optimized NL4-3 Rev sequence in place of the original Rev sequence. pCMVΔR9 (pHR2003) was a kind gift from Didier Trono (4).

RNA preparation for SHAPE and RRE gel migration experiments: RNAs were prepared by in vitro transcription using the MegaShortScript kit (Ambion/Life Technologies) according to manufacturers' recommendations. Transcription templates were generated by PCR from plasmids containing wt or mutant RREs using forward and reverse primers "5′ T7" (5'-GCTAATACGACTCACTATAGGGGAGCTTTGTTCCTTGGGTTC) and "3' SC" (5'- GAACCGGACCGAAGCCCGATTTGGATCCGGCGAACCGGATCGAAGGAGCTGTTGATCCT), respectively. The resulting amplicons contained wt or mutant short (232 nt) RRE-derived sequence flanked on the 3' end by a 45 nt structure cassette. RNAs were treated with Turbo DNase I for 1 hour at 37°C, heated at 85°C for 2 min and run on a denaturing gel (5% polyacrylamide (19:1)), 1x TBE, 7M urea) at constant temperature (45°C, 30W max). The RRE bands were excised, electroeluted at 200 V for 2 hours at 4°C, ethanol precipitated and stored at -20C in TE buffer (10 mM Tris, pH 7.6; 0.1 mM EDTA) prior to use.

RRE gel migration assay: Approximately 20 picomoles of RNA in a total volume of 5 µl renaturation buffer (10 mM Tris, pH 8.0, 100mM KCl, 0.1mM EDTA) was heated to 85°C and renatured by slow cooling (0.1°C/sec) to 25°C. Renatured RNA was incubated with 5 ul of 2X RNA folding buffer (70 mM Tris pH 8.0, 180 mM KCl, 0.3 mM EDTA, 10 mM $MgCl_2$, 5% of glycerol) at 37°C for 30 min and cooled to 4°C. The folded RRE was run on a native 8% polyacrylamide gel (29:1) for 22 hours at 4°C. Both gel and running buffer contained 1X TBE and 5mM $MgCl_2$ at the start of the run, and the running buffer was changed 1-2 times during the course of the run to maintain a constant ion distribution. After the gel run was complete, RNA bands were visualized by UV shadowing.

SHAPE: For in-gel SHAPE, 150 picomoles of wt RRE RNA in 20 µl renaturation buffer was heated and slow-cooled as described for the RRE gel migration assay above. After adding an equal volume of 2X RNA folding buffer, the reaction was mixed and incubated at 37°C for 30 min then cooled to 4°C. The completed folding reaction was split into equal, 20 µl portions, portions were loaded into adjacent, appropriately sized wells of an 8% native polyacrylamide gel and RNA conformers were fractionated by electrophoresis and visualized by UV shadowing as described for the RRE gel migration assay. "Fast-" and "slow-migrating" RRE conformer bands in adjacent lanes were excised from the gel and placed in 2 mL microfuge tubes designated "NMIA(+)" and "NMIA(-)", where they were incubated with 1 mL 1x TBE, 5 mM $MgCl_2$, 10% DMSO with or without 10 mM NMIA, respectively, at 37°C for 50 min . Following RNA modification, the gel slices were washed thrice with 1X TBE and the modified RNA was recovered from the gel by electroelution (200V, 2 hr, 4°C). For conventional SHAPE, the RNA was folded exactly as in the gel migration assay except that the final volume of the folded RNA mix was 150ul instead of 10ul and glycerol was excluded from the RNA folding buffer. Folded RNAs were divided into experimental (NMIA+) and control (NMIA-) aliquots (72 µl each), to which 8 µl 30 mM NMIA in anhydrous DMSO or DMSO alone was added, respectively. Modification reactions were incubated at 37°C for 50 min, then cooled to 4°C. For both in-gel and conventional SHAPE, treated RNAs were ethanol precipitated and re-suspended in 13ul nuclease free water. Subsequent reverse transcription of treated RNAs, cDNA processing/fractionation and SHAPE data analysis were conducted as previously described (5).

RNA preparation for Rev-RRE gel shift assay: Internally ^{32}P labeled 234 nt long RNAs were prepared by in-vitro transcription using T7 polymerase (NEB). To generate the template for the transcription, the RREs were PCR amplified using a primer set designed to introduce T7 promoter sequence at the 5' end of the RREs. The PCR products were cloned into the EcoRI site of pCR 2.1 TOPO TA vector (Invitrogen). Transcription templates were generated by linearizing the resulting recombinant plasmids by EcoRI digestion. Each transcription reaction contained (1X T7 RNA Pol buffer (NEB), 7.5 mM DTT, 20 Units of RNasin Plus Rnase Inhibitor (Promega), 0.5mM rNTP's (-UTP), 5mM of UTP, 50 uCi (0.84mM) of α32P UTP (Perkin Elmer), 40ng template DNA and 50U T7 RNA Pol in a volume of 20ul. The transcription reaction mixes were incubated at 37°C for 1 hour, treated with DNaseI (Promega), then incubated for an additional hour. RRE RNAs were heated at 85°C for 3 mins and immediately fractionated on a 4% polyacrylamide (28.5:1.5 mono:bis) gel containing 1X TBE and 5M urea. The bands corresponding to the 234nt transcription products were located by autoradiography and excised from the gel. RNAs were eluted from the gel slices by gently shaking in TE buffer overnight at 4°C, further purified by phenol/chloroform extraction and concentrated by ethanol precipitation. RNAs were then re-suspended in nuclease free dH2O, passed through a Chromaspin 30 column (Clontech) and stored at -80°C prior to use.

Rev-RRE gel-shift assay: To prepare the Rev-RRE complexes, two fold serial dilutions of Rev protein were made in Rev storage buffer (50mM Tris pH 8.0, 500mM NaCl, 1mM EDTA) immediately before use. 1 ul of each Rev dilution or 1 ul of Rev storage buffer alone was incubated with Rev binding buffer (10mM HEPES/KOH, pH7.8, 20mM KCL, 2mM MgCl2, 0.5mM EDTA, 1mMDTT, 10% glycerol, 5µg/ml yeast tRNA (Invitrogen) and 20U RNase inhibitor (Promega) in a volume of 10.6 ul on ice for at least 10 mins. Meanwhile, internally labeled RNA diluted to a final concentration of 1nM/µl was folded by incubating it in renaturation buffer (50mM NaCl, 10mM Hepes/KOH, pH 7.6, 2mM MgCl2) at 85°C for 3 min, then at room temperature for 15 mins. 1 ul of the renatured RNA mix was added to the preincubated Rev binding solutions. The resulting mixes were incubated on ice for 10 minutes before loading onto a 4% native polyacrylamide (28.5:1.5 mono:bis, 1X TBE) gel. Gels were run at 4°C, dried and exposed to a phosphorimaging screen for >16 hours and visualized with a Molecular Dynamics Phosphorimager and ImageQuant Software.

Rev dose response assay: 22-mm diameter wells of 12 well tissue culture plates were seeded with $2x10^5$ 293T cells in 2ml of culture medium (IMDM supplemented 10% Bovine Calf Serum, and 10µg/ml of Gentamycin). After twenty-four hours, cells in each well were co-transfected with 2µg pCMV_GagPol-RRE reporter plasmid and 2-fold increasing concentrations (0-32ng) of pCMV_Rev plasmid. The cells were also transfected with 100ng of secreted placental alkaline phosphatase (SEAP) plasmid as an internal control for transfection efficiency and with empty pCMV plasmid to equalize the amounts of DNA used in each transfection. Transfection was carried out using 3µl of Turbofect reagent (Thermo Scientific). Cell-free transfection supernatants were collected 48hours post transfection and the levels of p24 in the supernatants were determined (6). The SEAP levels in the supernatants were determined using SEAP kit (Applied Biosystems). The Rev dose response curves for Rev-RRE activity were generated by plotting the SEAP normalized p24 values against Rev plasmid concentration for each pCMV_GagPolRRE.

Hygromycin Resistance Assay: The transducing vector used in this assay was the Nef- version of a previously described pTR167 construct (3). The vector was created by deleting the entire 5491-bp region between the two NsiI sites located at nucleotides 1251 and 6742 of pNL4-3 (HIV NL4-3 [GenBank accession number M19921]). The deleted region includes part of *gag*, all of *pol*, and part of the *env* gene. A cassette containing the hygromycin resistance gene (Hyg^r, Hygromycin B phosphotransferase) driven by an SV40 early promoter–enhancer was inserted into the NheI site in the remaining *env* sequence at nucleotide 7520. This construct was rendered Nef- by deleting 97 nucleotides (nt 8789-8886) in the *nef* gene. This construct produces two species of mRNA: the Rev-RRE dependent genomic RNA and the Rev-RRE independent SV40 driven subgenomic Hyg^r mRNA.

Transducing viral stocks were produced in 293T cells. For this, $3.5x10^6$ 293T cells in 10ml of culture medium (IMDM supplemented 10% Bovine Calf Serum and 10µg/ml of Gentamycin) were seeded on 100 mm diameter tissue culture plates. After about 24 hours, cells were co-transfected with 20µg of pTR 167 Nef- w/ RRE and two helper plasmids, pCMVΔR9 (15µg) (4) and pCMV_VSV-G (5µg), using the calcium phosphate method. 48 hours post transfection, culture medium was harvested and spun at 3000rpm for 3 minutes at 4°C. Cell-free transfection supernatant (the viral stock) containing the transducing viruses were stored at -80°C for future use.

The viral stock was used to infect the target Hela cells. Twenty-four hours prior to infection, 60mm tissue culture plates were seeded with $5 x10^5$ Hela cells in 4ml of culture medium. Just before infection, the medium was sucked out of the plates and the cells were infected with 1ml of 10-fold serial dilutions of the viral stocks (prepared in the IMDM full medium). DEAE dextran was added to each plate at a concentration of 8µg/ml to facilitate viral adsorption. After 6 hours at 37°C, 3ml of fresh medium was added to each plate and the incubation was resumed. After 2 days of infection, the medium was replaced with fresh medium containing 200µg/ml of Hygromycin B. The hygromycin medium was changed after every 3rd day. After 14 days under hygromcyin selection, the cells were

washed twice with PBS then fixed and stained using 0.5% crystal violet in 50% methanol. The titer of hygromycin resistant colonies was determined.

Spreading Infections: The proviral constructs used in this assay contained the long RRE in the nef position of the previously described (1) pNL4-3 RRE(-) nef(-) backbone. The RRE in the normal position in the vector backbone was made non-functional by multiple third base silent mutations in gp41. The first 97 nt after the start codon of Nef was replaced by a polylinker rendering the vector nef-. The long RRE mutants and the long WT RRE were cloned into this polylinker site.

Transfection viral stocks were prepared by seeding 3 X 10^6 293T cells in a 75cm^2 flask a day prior to transfection. These cells were transfected with 5ug of the proviral DNA using the calcium phosphate method. Transfection supernatant was collected after 48hrs and was spun briefly at 2500Xg for 5 mins at 4°C to get rid of cell debris. SupT1 cells were infected with the transfection viral stocks with or without DEAE dextran. DEAE dextran mediated infections entailed adding 100ng p24 equivalent of viral stocks to 6X10^6 SupT1 cells in 1ml of culture medium (RPMI supplemented 10% fetal bovine serum, and 10ug/ml of gentamycin) containing 8µg/ml of DEAE. Infections were carried out in a loosely capped 50ml Falcon tube at 37°C. 6 hours after infection, the infected cultures were centrifuged at 2500Xg for 5 mins at 4°C and the supernatant was discarded. The infected cells were washed with 1X PBS twice and then gently suspended in 5ml of medium. The culture was then transferred to 25cm^2 flask for further incubation. The non-DEAE dextran infections were carried out by infecting 6X10^6 SupT1 cells in 10ml of culture medium with 300ng p24 equivalent of viral stocks in 25cm^2 flasks and then incubating the cultures at 37°C.

In both DEAE mediated and non-DEAE infections, after every 3-4th day, 2/3rd of the culture was replaced with fresh medium needed to maintain the culture volume at 5ml or 10ml, respectively. The replaced culture was spun at 2500Xg for 5 mins. at 4°C to remove the cell debris. The secreted p24 in the cell-free replaced culture was determined by ELISA to generate the growth curve.

The infection supernatants from the peak replication days were then used to infect fresh SupT1 cells. To carry out this infection, the TCID50 and thereby the MOIs of the infection viral stocks were determined as described (7) with p24 levels used as the measure of viral replication instead of RT activity. 6X10^6 SupT1 cells were then infected with 0.000005 MOI of the SupT1 passaged infection viral stocks (DEAE dextran method).

Growth Competition Assay: 6 X10^4 SupT1 cells were infected with 0.000005 MOI each of the two different SupT1 passaged viral stocks in 36 well plates and the assay was performed as described(7). However, the viral replication level was determined by measuring p24 in the cell-free culture medium instead of RT activity. The amount of proviral DNA from each culture was determined by heteroduplex tracking assay(7). For this, viral DNA was extracted using DNeasy blood and tissue DNA kit (Qiagen). The proviral DNA was amplified by nested PCR using outer forward oligo 2760 (5′ CGAGGATTGTGGAACTTCTGGG), outer reverse oligo 2761 (5′ GTGACTGGAAAACCCACCTC), inner forward oligo 2767 (5′ AATGACGCTGACGGTACAGGCCAGAC), and inner reverse oligo 2768 (5′ CAACTAGCATTCCAAGGCACAGCAGTGG). The final nested PCR product was 216nt long starting at nt 116 and ending at nt 331of the 351nt long RRE. The HTA probe amplicon was generated by PCR using wt proviral DNA as template and γ^{32}P end labeled oligo 2767 and the oligo 2768 as forward and reverse primers, respectively. The proviral DNA amplicons were annealed to the probe and run at 200V over 8% non-denaturing polyacrylamide gel at room temperature for 3 hours. The gel was dried and exposed to a phosphorimaging screen for >16 hours and visualized and quantified with a Molecular Dynamics Phosphorimager and ImageQuant Software.

1. Nasioulas G*, et al.* (1994) Elements distinct from human immunodeficiency virus type 1 splice sites are responsible for the Rev dependence of env mRNA. *J Virol* 68(5):2986-2993.
2. Srinivasakumar N*, et al.* (1997) The effect of viral regulatory protein expression on gene delivery by human immunodeficiency virus type 1 vectors produced in stable packaging cell lines. *J Virol* 71(8):5841-5848.
3. Rizvi TA & Panganiban AT (1993) Simian immunodeficiency virus RNA is efficiently encapsidated by human immunodeficiency virus type 1 particles. *J Virol* 67(5):2681-2688.
4. Naldini L*, et al.* (1996) In vivo gene delivery and stable transduction of nondividing cells by a lentiviral vector. *Science* 272(5259):263-267.
5. Lusvarghi S*, et al.* (2013) The HIV-2 Rev-response element: determining secondary structure and defining folding intermediates. *Nucleic acids research* 41(13):6637-6649.
6. Wehrly K & Chesebro B (1997) p24 antigen capture assay for quantification of human immunodeficiency virus using readily available inexpensive reagents. *Methods* 12(4):288-293.
7. Abraha A, Troyer RM, Quinones-Mateu ME, & Arts EJ (2005) Methods to determine HIV-1 ex vivo fitness. *Methods Mol Biol* 304:355-368.
